# Comparison of Vonoprazan Triple Therapy, Bismuth Quadruple Therapy, and Amoxicillin Therapy for Helicobacter pylori Infection: A Systematic Review

**DOI:** 10.7759/cureus.83142

**Published:** 2025-04-28

**Authors:** Zafeer-ul-Hassan Iqbal, Syed Muhammad Hassan Bukhari, Shama Shahid Rana, Rana Asjad Dilshad, Amna Saeed, Mars Christian Aragon Sta Ines, Ali Haroon

**Affiliations:** 1 Internal Medicine, Countess of Chester Hospital, Chester, GBR; 2 Internal Medicine, Sunderland Royal Hospital, Sunderland, GBR; 3 Medicine, King's College Hospital, London, GBR; 4 Medicine, Social Security Basic Health Unit, Punjab Employees Social Security Institution (PESSI), Lahore, PAK; 5 General Medicine, Nishtar Medical University, Multan, PAK; 6 Internal Medicine, Shaukat Khanum Memorial Cancer Hospital and Research Centre, Lahore, PAK; 7 Medical Education and Emergency Medicine, University Hospital Coventry, Coventry, GBR; 8 Gastroenterology and Hepatology, Hamdard University Hospital, Karachi, PAK

**Keywords:** amoxicillin therapy, bismuth-quadruple therapy, drug therapy, helicobacter pylori infection, vonoprazan triple therapy

## Abstract

*Helicobacter pylori* (*H. pylori*) infection is a significant global health concern, leading to life-threatening gastric disorders. Despite innovation in standardised treatment protocols, it faces challenges due to rising antibiotic resistance, creating the need for alternative treatments. There is a need to synthesize evidence from recent trials to enhance knowledge in critical care practice. This review synthesizes current trial data to strengthen the understanding of the literature. This systematic review was conducted according to the Preferred Reporting Items for Systematic Reviews and Meta-Analyses (PRISMA) principles. The literature was searched using text terms and controlled vocabulary, employing Boolean operators "AND," "OR," and various combinations across PubMed, Embase, and the Cochrane Library. Open-access, full-text English papers from 2014 to 2024 involving human-based studies were searched. The quality was assessed using Revised Cochrane Risk of Bias Tool for Randomized Trials, Version 2.0* (*ROB 2.0), and the evidence was appraised using Grading of Recommendations Assessment, Development and Evaluation (GRADE). A total of 68 articles were retrieved through the initial search. After screening and verifying eligibility according to the pre-specified inclusion criteria, the methodological quality of 25 randomized controlled trials (RCTs) was assessed using the Cochrane Risk of Bias 2.0 tool. The GRADE tool categorised three high-ROB RCTs as "low quality." However, four RCTs had low ROB and were classified as "high quality." Eighteen RCTs had uncertain ROB, lowering the evidence by one point to "moderate quality." The study found that bismuth-quadruple therapy is an excellent first-line treatment for *H. pylori* infection, although its adverse effects restrict its use. Vonoprazan Triple Therapy offers a superior eradication rate with improved tolerability, making it an effective treatment option. However, high doses of amoxicillin emerge as the safest and most effective treatment, especially where clarithromycin resistance is prevalent. The management of *H. pylori* infection is a dynamic process. As resistance patterns evolve and new treatments are developed, it is important for healthcare providers to stay informed about emerging options. Personalized treatment, taking into account patient preferences, tolerability, and concerns about resistance, will likely continue to offer the best chance for successful eradication. Ultimately, the goal is to select the most suitable therapy for each patient, ensuring both the effectiveness and comfort of the treatment.

## Introduction and background

Globally, *Helicobacter pylori* (*H. pylori*) is one of the most widespread bacterial infections [1]. More than 50% of the population is affected [2]. Low-income and underdeveloped nations have *H. pylori* infection rates of up to 80%, whereas high-income nations report rates ranging from 20% to 50% [3]. Marshall and Warren discovered *H. pylori* as a pathogen in the early 1980s, which opened the door to more complex research [4]. The efficacy and frequency of *H. pylori* infection depend on the genetic susceptibility of both humans and bacteria [5].

Although *H. pylori* primarily causes gastrointestinal disorders, it may also lead to neurological, dermatological, hematological, ophthalmic, cardiovascular, metabolic, hepatobiliary, or allergic issues [6]. *H. pylori* was classified as a class I carcinogen by the WHO in 1994 [7].

Females have higher rates of antibiotic resistance in *H. pylori* infection than males. Resistance levels of *H. pylori* bacteria against antibiotics are greater in female patients because of various factors. The actions of estrogen hormones influence how both the immune system and the gastric environment affect microbial responses. The development of bacterial resistance is accelerated when patients are prescribed more antibiotics due to increased healthcare involvement. *H. pylori* survives and adapts better during treatment in male and female bodies due to unique gastric pH levels and microbiome compositions [8]. Molecular mechanisms include single-drug resistance related to mutational changes and enzymatic degradation of antibiotics. Multidrug resistance includes increased efflux pump expression, resulting in decreased drug concentration. The heterogeneous resistance mechanism includes adaptive resistance [9,10]. *H. pylori* infection exhibits a wide disease spectrum, ranging from asymptomatic gastritis and peptic ulcer disease to gastric cancer [11].

Vonoprazan (VPZ), a potassium-competitive acid blocker, suppresses stomach acid quickly and effectively, increasing *H. pylori* eradication [12]. The US FDA has authorised VPZ in conjunction with amoxicillin or clarithromycin to treat adult *H. pylori* infection [13]. Within potassium-competitive acid blockers (P-CABs) medications, VPZ proves superior to traditional proton pump inhibitors (PPIs) for the treatment of *H. pylori* infections. Medical practitioners prefer VPZ over PPIs such as omeprazole due to its consistent acid suppression function. Patients need to take VPZ for a period of 7 to 14 days when paired with clarithromycin and amoxicillin antibiotic treatments. VPZ offers superior therapeutic capabilities that position it well for treating difficult-to-eradicate infections. VPZ-based triple treatment achieves a 90% eradication rate [14]. The FDA approves VPZ for the eradication of *H. pylori*. Due to antibiotic overuse and strain variances in China, Chinese guidelines suggest a 14-day therapy; however, the treatment administered lasted 7 days [7].

Bismuth salt, PPI, and two antibiotics (tetracycline and metronidazole) form a bismuth-based quadruple treatment [15]. First-line therapy for children with *H. pylori* infection is bismuth-based triple treatments, according to ESPGHAN/NASPGHAN (ESPGHAN stands for European Society for Pediatric Gastroenterology, Hepatology, and Nutrition & NASPGHAN stands for North American Society for Pediatric Gastroenterology, Hepatology, and Nutrition) guidelines [16]. Its use offers the following advantages: a strong bacteriostatic effect that is not altered by resistance and beneficial synergy when combined with several antibiotics, making it possible to overcome bacterial resistance [17].

Amoxicillin, which works against *H. pylori* but is less effective during stationary growth, has time-dependent bactericidal action. Amoxicillin dose influences *H. pylori* eradication [18,19]. *H. pylori* produces β-lactamase and exhibits high-level resistance to amoxicillin. Minor amoxicillin resistance is linked to a pbp1A point mutation [20].

*H. pylori* infection is a significant global health concern that leads to gastrointestinal problems and an increased risk of lymphoma, necessitating effective eradication strategies. However, antibiotic resistance and patient compliance pose challenges. VPZ provides potent acid suppression. Bismuth-Quadruple Therapy (BQT) excels in resistant strains, especially in clarithromycin-resistant cases. Amoxicillin reduces antibiotic use and works in metronidazole-resistant cases. Vonoprazan Triple Therapy (VTT), BQT, and Amoxicillin Therapy emerged as key treatment options. The treatment selection included VTT, BQT, and Amoxicillin because these medications prove effective against *H. pylori* infections, except those showing antibiotic resistance. The superior acid-blocking power of VPZ allows it to increase the effectiveness of the antibiotics clarithromycin and amoxicillin. A combination of bismuth substances along with multiple antibiotics in BQT shows effectiveness against *H. pylori* strains that are resistant to clarithromycin. The medical community continues to rely on amoxicillin because manifestations of resistance to the drug remain minimal. The prescribed treatment strategies combine to ensure the best possible elimination of *H. pylori* bacteria while fighting against antibiotic resistance across different areas. However, a gap exists regarding their comparative effectiveness, long-term success, and adverse effects. Previous research investigates VTT, BQT, and Amoxicillin treatments while assessing effectiveness, long-term results, and reported side effects but does not yield a single conclusion. VTT produces superior acid suppression that leads to better eradication rates in areas with high clarithromycin resistance, but its extended success and reinfection risk assessment is ongoing. The effectiveness of BQT against *H. pylori* strains resistant to clarithromycin exists, yet patient adherence problems remain because the treatment duration is long, and symptoms include nausea and black stool appearance. The use of amoxicillin continues as a foundational drug in triple therapy because resistance is minimal and the drug provides excellent safety properties, although its effectiveness decreases against multiple organisms. The medical community requires further studies that evaluate both the long-term success of such treatments and their adverse effects, as well as their relative effectiveness while taking ethnicity patterns and resistance mechanisms into account. A systematic review evaluating this outcome provides insight for optimizing *H. pylori* treatment strategies.

## Review

Methodology

This systematic review was conducted in accordance with the PRISMA guidelines [21]. The research question was formulated using the PICO framework, as described in Table 1 [22].

**Table 1:** PICO framework.

Concepts	Text Words	Controlled Vocabulary
Population/Problem	“Adult patients”, “H. pylori”, “Helicobacter infection”	"Helicobacter pylori"[Mesh]
Intervention	“Triple Therapy”, “Vonoprazan”	"Vonoprazan", "Acid Suppressants"[Mesh]
Comparison	“Bismuth”, “Quadruple Therapy”, “Amoxicillin”	"Bismuth Quadruple Therapy"[Mesh], "Amoxicillin"[Mesh]
Outcomes	“Eradication rate”, “Treatment success”, “Side effects”, “Patient compliance”	"Microbial Sensitivity Tests"[Mesh], "Adverse Drug Reaction Reporting Systems"[Mesh], "Patient Compliance"[Mesh]

Research question

What are the comparative effects of Vonoprazan Triple Therapy (VTT), Bismuth-Quadruple Therapy (BQT), and Amoxicillin Therapy for Helicobacter pylori infection?

Search strategy and search terms

Searches were conducted using the terms "Vonoprazan Triple Therapy," "Bismuth-Quadruple Therapy," "Amoxicillin Therapy," "Helicobacter pylori infection," "Uses," "Safety profile," "Adverse reaction," "Eradication rate," "Adherence," and "Compliance." Both text words and controlled vocabulary were used, employing Boolean operators "AND," "OR," and various combinations on PubMed, Embase, and Cochrane. Limiters were applied to search for open-access, full-text, English-language papers from 2014 to 2024 related to human studies.

Search string

("Helicobacter pylori" OR "H. pylori") AND ("antibiotic resistance" OR "drug resistance" OR "treatment failure") AND ("Vonoprazan" OR "Bismuth-Quadruple Therapy" OR "Amoxicillin") AND ("long-term success" OR "adverse effects" OR "comparative effectiveness") AND ("2014"[Date - Publication] : "2024"[Date - Publication]) AND ("full text"[Filter] AND "English"[Language] AND "open access"[Filter]).

Inclusion criteria

In this review, only experimental studies (randomized controlled trials, RCTs) were included, with varying sample sizes. Studies in which adult patients of either gender underwent VTT, Bismuth Quadruple Therapy, or Amoxicillin Therapy for Helicobacter pylori infection were included. The RCTs focused on efficacy, patient compliance, and adverse reactions to VTT, BQT, or Amoxicillin Therapy. Studies from the last decade that were open-access, written in English, and had full-text availability were included in the synthesis.

Exclusion criteria

The review excluded all other study designs, such as cohort, case-control, observational studies, case reports, case series, conference abstracts, editorials, letters, review papers, and meta-analyses. Studies on teenagers, children, and animals were also excluded. Additionally, studies in which patient outcomes were not related to VTT, BQT, or Amoxicillin Therapy-associated complications, such as efficacy, patient compliance, and adverse reactions, conducted before 2014 were excluded due to restricted data access, incomplete analysis, and payment issues.

Study selection process

Initial screening included two independent reviewers reading the articles’ titles and abstracts. Subsequently, the two reviewers conducted a full-text review by comprehensively reading the articles. In case of any disagreement between reviewers, a consensus was developed [23]. The review included only those studies that were available in full text and met the inclusion criteria.

Methodological quality assessment

The Cochrane Risk of Bias 2.0 tool was used to assess the risk of bias, categorizing studies into high, low, and some concerns regarding the risk of bias [24]. The Grading of Recommendations Assessment, Development, and Evaluation (GRADE) was used to determine the strength of trial recommendations, categorized as high quality, moderate quality, low quality, and very low quality [25].

Data extraction and synthesis

A datasheet was created to collect details from the included studies for the synthesis of study findings. For this study, it encompassed basic information such as study design, demographic characteristics, and characteristics related to the outcomes of interest, including patient adherence and compliance, side effects of specific therapies, and information about the efficacy of particular treatments, such as eradication rates. After data collection, a thematic analysis using an inductive, data-driven approach was employed to analyze the datasheet [26]. Then, an iterative approach was applied for a more in-depth study and convergence of the results [27]. Studies were critically analyzed to synthesize the evidence, ensuring the practice is evidence-based.

Ethical consideration

**Figure 1:**
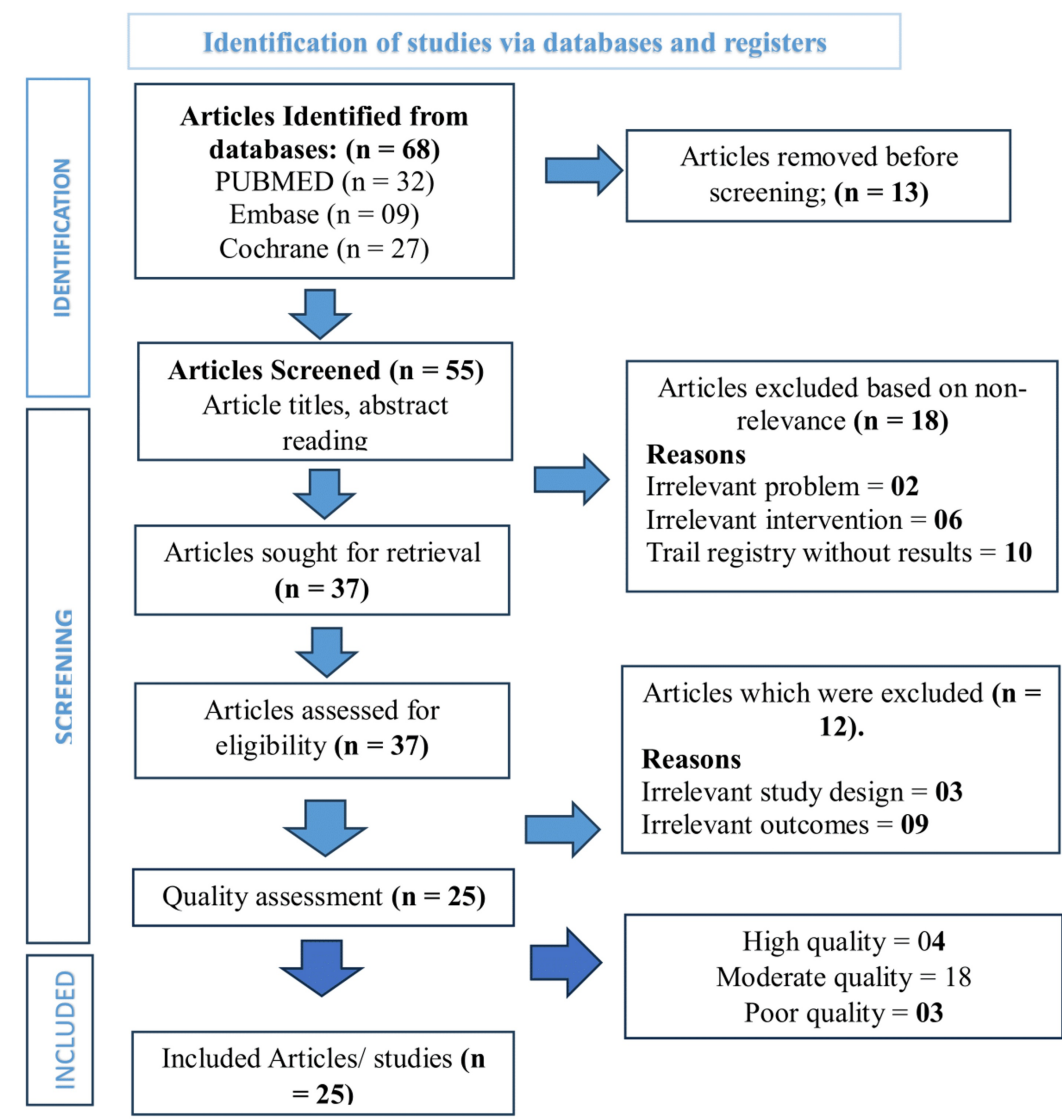
PRISMA flow chart. PRISMA: Preferred Reporting Items for Systematic Reviews and Meta-Analyses.

The review adhered to the Helsinki Declaration to ensure that ethical standards were met throughout the study. There are no conflicts of interest among the reviewers. The study was conducted using specific keywords to ensure reproducibility. It will be published in a medical journal to disseminate the findings publicly while ensuring confidentiality and anonymity. The study met the PRISMA guidelines (Figure 1).

Results

The PRISMA guidelines were followed to systematically synthesize the evidence in this review. A total of 68 articles were retrieved during the initial search using keywords, text words, and controlled vocabulary on PubMed, Embase, and the Cochrane Library databases. Thirteen duplicate articles were removed using EndNote. Then, 55 articles were selected for screening. Eighteen irrelevant articles were removed based on title and abstract screening. The eligibility of 37 articles was determined through pre-specified criteria. Furthermore, 12 irrelevant articles were excluded through a thorough and in-depth review. After the eligibility check, only 25 articles were selected for quality assessment.

Cochrane risk of bias assessment of studies included in the systematic review

Table 2 presents the Cochrane Risk of Bias Assessment for studies in the systematic review. This assessment evaluates potential biases across key domains, including selection, performance, detection, and reporting. It provides a comprehensive overview of the methodological quality of the studies, highlighting areas of low, high, or unclear risk of bias. The results inform the overall confidence in the review’s findings and the reliability of the evidence presented.

**Table 2:** Cochrane risk of bias assessment of studies included in the systematic review.

Author/Year	Randomisation Process	Deviations from Intended Intervention	Missing Outcome Data	Outcome Measurement	Selection of Reported Result	Overall Quality (RoB)
Chey et al., 2022 [28]	Low	Some concerns	Low	Low	Low	Moderate
Murakami et al., 2016 [29]	Low	Low	Some concerns	Low	Low	Moderate
Chen et al., 2023 [30]	Low	Some concerns	Low	Some concerns	Low	Moderate
Waqar et al., 2023 [31]	Low	Low	Low	Low	Some concerns	Moderate
Cheung et al., 2024 [32]	Low	Low	Some concerns	Low	Low	Moderate
Bunchorntavakul et al., 2021 [33]	Low	Some concerns	Low	Some concerns	Low	Moderate
Ang et al., 2022 [34]	Some concerns	Some concerns	Low	Some concerns	Some concerns	Poor
Waqar et al., 2024 [35]	Low	Low	Some concerns	Low	Low	Moderate
Ratana-Amornpin et al., 2023 [36]	Low	Low	Low	Low	Low	High
Kakiuchi et al., 2020 [37]	Low	Some concerns	Some concerns	Low	Some concerns	Poor
Liang et al., 2024 [38]	Low	Low	Low	Low	Low	High
Chiu et al., 2024 [39]	Low	Some concerns	Low	Low	Some concerns	Moderate
Sue et al., 2018 [40]	Low	Low	Some concerns	Low	Low	Moderate
Sakurai et al., 2016 [41]	Low	Low	Some concerns	Low	Low	Moderate
Chen et al., 2024 [42]	Low	Low	Low	Low	Low	High
Hsu et al., 2023 [43]	Low	Some concerns	Low	Low	Some concerns	Moderate
Koroglu et al., 2022 [44]	Low	Low	Low	Low	Some concerns	Moderate
Zhao et al., 2024 [45]	Low	Low	Some concerns	Low	Low	Moderate
Macedo et al., 2023 [46]	Low	Low	Low	Some concerns	Low	Moderate
Wang et al., 2021 [47]	Low	Low	Some concerns	Low	Low	Moderate
Huang et al., 2024 [48]	Low	Low	Some concerns	Low	Low	Moderate
Yan et al., 2024 [49]	Low	Low	Low	Some concerns	Low	Moderate
Hu et al., 2022 [50]	Low	Some concerns	Low	Low	Low	Moderate
Qiu et al., 2024 [51]	Low	Some concerns	Some concerns	Low	Low	Poor
Jiang et al., 2024 [52]	Low	Low	Low	Low	Low	High

Twenty-five RCTs were analyzed for methodological quality, with three categorized as having high ROB. The GRADE tool categorized these three high ROB RCTs as “low quality.” However, four RCTs had low ROB and were classified as “high quality.” Eighteen RCTs had uncertain ROB, lowering the evidence by one point to “moderate quality.”

Characteristics and findings of studies included in the review

Table 3 describes the characteristics and findings of the studies included in the review. It encompasses study design, sample size, interventional protocol, outcomes measured, findings, comparative effectiveness, complications, and challenges.

**Table 3:** Characteristics and findings of studies included in the review.

Author/Year	Study Design	Sample Size	Interventional Protocol	Outcome Measured	Findings	Comparative Effectiveness	Complications	Challenges
Chey et al., 2022 [28]	Randomized Controlled Trial	1,046 patients	Dual: 20 mg vonoprazan BID + 1 g amoxicillin TID; Triple: 20 mg vonoprazan BID + 1 g amoxicillin + 500 mg clarithromycin BID; Triple lansoprazole: 30 mg BID + 1 g amoxicillin + 500 mg clarithromycin BID	Eradication rates in non-resistant strains and clarithromycin-resistant infections	- Vonoprazan triple therapy eradication rate: 84.7% (non-resistant) - Vonoprazan dual therapy eradication rate: 78.5% (non-resistant) - Lansoprazole triple therapy eradication rate: 78.8% (non-resistant)	Vonoprazan outperformed in clarithromycin-resistant strains (65.8%–69.6% vs. 31.9%). Vonoprazan regimens outperformed lansoprazole-based treatment (80.8%, 77.2% vs. 68.5%).	Not mentioned	Not mentioned
Murakami et al., 2016 [29]	Randomized Controlled Trial	650 subjects	First-line: Vonoprazan 20 mg vs. Lansoprazole 30 mg + Amoxicillin 750 mg + Clarithromycin 200/400 mg; Second-line (open-label): Vonoprazan 20 mg + Amoxicillin 750 mg + Metronidazole 250 mg (for 50 patients with failed first-line and satisfactory compliance)	Eradication rates of H. pylori, safety, and tolerability	- Vonoprazan: 92.6% - Lansoprazole: 75.9% primary eradication - 16.7% higher efficacy in favor of vonoprazan (p < 0.0001) - Second-line eradication: 98.0% - No significant differences in tolerability between treatments	Vonoprazan was significantly more effective than lansoprazole in first-line therapy, confirming non-inferiority	No notable differences in tolerability or safety between groups	Patient adherence, antibiotic resistance, and generalizability of findings
Chen et al., 2023 [30]	Randomized Controlled Trial (RCT)	300	Group A: Berberine 500 mg + Amoxicillin 1000 mg + Vonoprazan 20 mg; Group B: Vonoprazan 20 mg + Amoxicillin 1000 mg + Clarithromycin 500 mg + Colloidal Bismuth Tartrate 220 mg; Group C: Rabeprazole 10 mg + Amoxicillin 1000 mg + Clarithromycin 500 mg + Colloidal Bismuth Tartrate 220 mg; (All BID for 14 days)	Eradication rate, symptom improvement, patient compliance, and adverse event incidence	- ITT eradication: A: 70.0%, B: 77.0%, C: 69.0% - PP eradication: A: 81.4%, B: 86.5%, C: 78.4% - No statistically significant differences (P > 0.05)	No significant differences in compliance, symptom improvement, or eradication rates among groups	Similar adverse reaction rates across groups (P > 0.05)	Not mentioned
Waqar et al., 2023 [31]	Randomized Controlled Trial	122 patients	Esomeprazole group (14 days): Esomeprazole 20 mg BID + Amoxicillin 1000 mg BID + Levofloxacin 500 mg QD; Vonoprazan group (7 days): Vonoprazan 20 mg BID + Amoxicillin 1000 mg BID + Levofloxacin 500 mg QD	Eradication rate, compliance, and cost-effectiveness	- Eradication rate: Vonoprazan 95.1%, Esomeprazole 93.1% (p = 0.64) - Compliance: Esomeprazole 95% (p = 0.07) - Cost-effectiveness: Vonoprazan had a lower cost (731.8 PKR)	Vonoprazan showed slightly higher eradication, better tolerability, and greater cost-effectiveness due to shorter treatment duration	Not mentioned	Not mentioned
Cheung et al., 2024 [32]	Randomized Controlled Trial	298 subjects	Three 14-day regimens: 1) VA-dual: Vonoprazan 20 mg BID + Amoxicillin 1 g TID; 2) VAC-triple: Vonoprazan 20 mg BID + Amoxicillin 1 g + Clarithromycin 500 mg BID; 3) Bismuth-quadruple: Esomeprazole + Tetracycline + Metronidazole	Eradication rate of H. pylori (ITT & PP analysis)	- ITT: VA-dual (96.0%), VAC-triple (95.9%), Bismuth-quadruple (92.0%) - PP: VA-dual (96.7%), VAC-triple (97.4%) - Statistically significant non-inferiority (p = 0.009, 0.010)	VA-dual and VAC-triple therapies were comparable to bismuth-quadruple therapy with fewer side effects	Adverse event rates: - VA-dual: 39.0% - VAC-triple: 56.1% - Bismuth-quadruple: 71.0%	High resistance rates to clarithromycin and levofloxacin (>30%) in Southern China
Bunchorntavakul et al., 2021 [33]	Randomized Controlled Trial	122 (61 per group)	7-VAC: Vonoprazan 20 mg BID + Amoxicillin 1000 mg BID + Clarithromycin 500 mg BID for 7 days; 14-OAC: Omeprazole 20 mg BID + Amoxicillin 1000 mg BID + Clarithromycin 500 mg BID for 14 days	H. pylori eradication rates	7-VAC: 96.7% (ITT), 98.3% (PP); 14-OAC: 88.5% (ITT), 93.1% (PP); P-values: 0.083 (ITT), 0.159 (PP)	7-VAC showed a trend toward higher eradication rates but was not statistically significant compared to 14-OAC.	Bitter taste, nausea, dizziness	No major challenges were reported
Ang et al., 2022 [34]	Randomized Controlled Trial	244	Vonoprazan-based: 7 days of Amoxicillin 1 g + Clarithromycin 500 mg + Vonoprazan 20 mg BID; PPI-based: 14 days of Amoxicillin 1 g + Clarithromycin 500 mg + Omeprazole/Esomeprazole/Rabeprazole 20 mg BID	H. pylori eradication success rates; treatment failure prediction using clarithromycin resistance	Vonoprazan-based ITT eradication: 87.4%; PPI-based: 88.0%. Vonoprazan-based PP eradication: 96.3%; PPI-based: 94.0%. Clarithromycin resistance predicted treatment failure (RR 11.4, p = 0.025).	Vonoprazan-based triple therapy was non-inferior to PPI-based triple therapy.	No significant differences in adverse events between groups	Clarithromycin resistance impacted treatment success
Waqar et al., 2024 [35]	Randomized Controlled Trial	122 patients	EAL group: Esomeprazole 20 mg BID + Amoxicillin 1000 mg BID + Levofloxacin 500 mg QD for 2 weeks; VAL group: Vonoprazan 20 mg BID + Amoxicillin 1000 mg BID + Levofloxacin 500 mg QD for 1 week	H. pylori eradication rate; drug safety (adverse effects)	Eradication Rate: EAL: 93.4% (57/61); VAL: 95% (58/61). Adverse effects more common in EAL group: Nausea (23%), Bitter taste (67.2%), Abdominal pain (26.2%), Headache (32.8%)	Vonoprazan-based therapy (VAL) showed slightly higher eradication and fewer adverse effects than Esomeprazole-based therapy (EAL).	More adverse effects in EAL: nausea, bitter taste, abdominal pain, headache.	Patient compliance and therapy duration; shorter duration favored in Vonoprazan group
Ratana-Amornpin et al., 2023 [36]	Randomized Controlled Trial	100 patients	Four groups: 1) 14-day dual: Amoxicillin 500 mg OD + Vonoprazan 20 mg BID; 2) 14-day triple: Amoxicillin 1 g BID + Clarithromycin-MR 1 g OD + Vonoprazan 20 mg OD; 3) 7-day high-dose: Amoxicillin 1 g BID + Clarithromycin-MR 1 g OD + Vonoprazan 60 mg OD; 4) 14-day triple + bismuth: Amoxicillin 1 g BID + Clarithromycin-MR 1 g OD + Vonoprazan 20 mg BID + Bismuth subsalicylate 1048 mg BID	H. pylori eradication rate	14-day dual therapy: 66.7% (14/21); 14-day triple: 59.3% (16/27); 7-day high-dose vonoprazan triple: 92.3% (24/26); 14-day vonoprazan triple + bismuth: 96.2% (25/26)	Higher doses of vonoprazan and/or addition of bismuth improved eradication rates significantly.	Not mentioned	14-day dual/triple vonoprazan regimens were ineffective alone; higher doses or bismuth needed
Kakiuchi et al., 2020 [37]	Randomized Controlled Trial	Not specified	Group 1 (BFR–): VPZ 20 mg BID + Amoxicillin 750 mg BID + Clarithromycin 400 mg BID for 7 days; Group 2 (BFR+): Same regimen + BFR (3 tablets/day) for 7 days	Gut microbiota diversity (α-diversity, β-diversity), stool consistency, diarrhoea incidence	BFR+ group preserved α-diversity; β-diversity comparable across groups. Diarrhoea: BFR+ (56.5%) vs BFR− (73.1%), not significant (P = .361). Stool consistency also not significantly different (P = .415).	BFR improved microbial diversity and reduced stool softening vs. VPZ alone.	Higher diarrhoea incidence in BFR− group, not statistically significant.	No significant reduction in diarrhoea; small sample size.
Liang et al., 2024 [38]	Randomized Controlled Trial	600 patients	Group A: Vonoprazan 20 mg + Amoxicillin 750 mg + Bismuth potassium citrate 220 mg for 14 days; Group B: Standard quadruple therapy: Esomeprazole, Clarithromycin, Amoxicillin, Bismuth	H. pylori eradication, side effects, cost	ITT eradication: Group A: 83.7%, Group B: 83.2%; PP: A: 90.9%, B: 89.7%. Fewer side effects in A (13.7% vs 28.6%, P < 0.001). Bitter mouth: A (3.7%) vs B (16.2%, P < 0.001).	Similar eradication; fewer adverse effects in Group A.	Fewer side effects in Group A vs Group B.	Antibiotic resistance and adverse effects remain concerns.
Chiu et al., 2024 [39]	Randomized Controlled Trial	628 patients	7-day vonoprazan triple therapy with high-dose amoxicillin (VAC-7) vs. 14-day extended sequential therapy (S-14)	Eradication rate (primary endpoint); adverse effect rates and compliance (secondary outcomes)	Eradication rates: VAC-7 (88.6% PP / 81.8% ITT) vs. S-14 (90.3% PP / 81.4% ITT); VAC-7 was non-inferior to S-14 in ITT analysis; Fewer adverse effects in VAC-7 (nausea, anorexia, dizziness, fatigue, severe AEs); Higher compliance in VAC-7 (94% adherence)	VAC-7 was non-inferior to S-14 in ITT analysis	Fewer incidences of nausea, anorexia, dizziness, fatigue, and severe adverse events in VAC-7	Not mentioned
Sue et al., 2018 [40]	Randomized Controlled Trial	147 (41 CAM-resistant, 106 CAM-susceptible)	V-AC: Vonoprazan 20 mg BID + Amoxicillin 750 mg BID + Clarithromycin 200/400 mg BID; PPI-AC: Lansoprazole 30 mg, Rabeprazole 10 mg, or Esomeprazole 20 mg BID + Amoxicillin 750 mg BID + Clarithromycin 200/400 mg BID	H. pylori eradication rates (ITT and PP); safety evaluation	V-AC in CAM-susceptible: 87.3% (ITT), 88.9% (PP); PPI-AC: 76.5% (ITT), 86.7% (PP); No significant difference (P = .21 ITT, P = .77 PP); V-AC in CAM-resistant: 82.9%	No significant difference between V-AC and PPI-AC in CAM-susceptible individuals	Not specifically reported	V-AC eradication rate <90% in CAM-susceptible patients
Sakurai et al., 2016 [41]	Randomized Controlled Trial	24 (12 per group)	Four treatment sequences: Vonoprazan + Amoxicillin + Clarithromycin or Metronidazole, with 7–14 day washouts	Safety and tolerability	Vonoprazan-amoxicillin-clarithromycin increased plasma vonoprazan and clarithromycin exposure; vonoprazan-amoxicillin-metronidazole did not affect VPZ/metronidazole PK; Amoxicillin PK unaffected	Vonoprazan-amoxicillin-clarithromycin increased exposure, metronidazole combo did not	Seven AEs reported; 2 discontinued due to rash and liver function abnormalities	Not mentioned
Chen et al., 2024 [42]	Randomized Controlled Trial	78 patients (41 HDDT, 37 BQT)	Comparison of HDDT vs. BQT for H. pylori eradication	Changes in gut microbiota diversity and composition	HDDT caused milder disturbance; alpha diversity rebounded in HDDT group	HDDT had milder gut microbiota impact vs. BQT	Increased Klebsiella, Escherichia fergusonii, Enterococcus faecium, E. faecalis (BQT group)	Persistent Proteobacteria dominance after eradication
Hsu et al., 2023 [43]	Randomized Controlled Trial	918 patients (306/group)	1) 14-day Hybrid Therapy; 2) 14-day High-Dose Dual Therapy; 3) 10-day Bismuth Quadruple Therapy	H. pylori eradication rate	14-day Hybrid Therapy: 91.5%; 14-day High-Dose Dual: 83.3%; 10-day Bismuth Quadruple: 90.2%	Hybrid and Bismuth Quadruple superior to High-Dose Dual, but similar to each other	Hybrid: 27%, High-Dose Dual: 13%, Bismuth Quadruple: 32% adverse events	Balancing efficacy and safety, high-efficacy therapies had more side effects
Koroglu et al., 2022 [44]	Randomized Controlled Trial	303 patients	Groups: sTT (n=76), BQT (n=78), ST (n=75), HT (n=74) for H. pylori eradication	H. pylori eradication rate (ITT & PP)	HT had highest eradication rate, sTT had lowest	HT significantly better than sTT (P = 0.028 ITT, P = 0.004 PP)	Not mentioned	Antibiotic resistance, low eradication with sTT
Zhao et al., 2024 [45]	Randomized Controlled Trial	30 patients	14-day BQT with Amoxicillin + Clarithromycin, followed by one-time fecal microbiota transplant (FMT) or placebo	Gut microbiota changes (alpha-diversity, genus/phylum, resistance genes), GI symptoms (GSRS)	BQT reduced beneficial bacteria, increased pathogens/resistance genes; baseline return by week 10; FMT improved symptoms, not microbiota	FMT group had lower GSRS total and diarrhoea scores at week 3 vs. placebo	Not mentioned	Short-term microbiota disruption from BQT
Macedo et al., 2023 [46]	Randomized Controlled Trial	100 patients (54% women, mean age 55 ±14)	BQT: Bismuth 140 mg + Metronidazole 125 mg + Tetracycline 125 mg, QID ×10 days + Esomeprazole 40 mg BID; vs. HDADT: Amoxicillin alternating 1000/500 mg QID ×14 days + Esomeprazole 40 mg BID	H. pylori eradication, symptoms, tolerability	HDADT > BQT: ITT 96.2% vs. 81.4% (p = .022); PP 95.9% vs. 81.0% (p = .025); Second-line: HDADT 100% vs. BQT 62.5% (p = .028)	HDADT more effective, especially in second-line therapy	Similar side effects: ITT 7.0% vs. 2.0% (p = .254); PP 4.8% vs. 0% (p = .210)	Cost of BQT and lower tolerability
Wang et al., 2021 [47]	Randomized Controlled Trial	116 (tetracycline group), 168 (furazolidone group)	Bismuth quadruple therapy with either tetracycline 1.0 g or furazolidone 0.1 g + PPI + Colloidal pectin bismuth + Amoxicillin for 12 days	H. pylori eradication rate	Tetracycline group: 92.7% (PP), 87.1% (ITT); Furazolidone group: 89.8% (PP), 83.9% (ITT)	No significant difference in eradication rates (P > 0.05)	Adverse events: 20.2% (tetracycline) vs. 37.6% (furazolidone), P = 0.003	Not mentioned
Huang et al., 2024 [48]	Randomized Controlled Trial	Not specified (conducted at 23 centres, Fujian, China)	Regimens: BQT, BQT-V, VAT-7, VAT-10, VAT-14	Eradication rate, adverse events	VAT-10 (93.2%) and VAT-14 (92.2%) had higher eradication rates than BQT (80.2%)	VAT-10 and VAT-14 showed superior eradication compared to BQT (P = 0.022 and P = 0.046)	Adverse event incidence was lower in VAT-10 (25.27%) and VAT-14 (13.73%) compared to BQT (37.62%)	VAT-7 and BQT-V were terminated early due to low eradication rates
Yan et al., 2024 [49]	Randomized Controlled Trial	314 patients	VA-dual: 10-day Vonoprazan-Amoxicillin; Bismuth-quadruple: 14-day therapy	H. pylori eradication rates, incidence of adverse events, compliance rates	VA-dual: 86.0% (ITT), 88.2% (mITT), 90.8% (PP); B-quadruple: 89.2% (ITT), 91.5% (mITT), 91.3% (PP); VA-dual had significantly fewer adverse events (P < 0.001); Poor compliance contributed to eradication failure (P < 0.001)	VA-dual showed similar efficacy to B-quadruple with fewer adverse events	VA-dual group had fewer adverse events; poor compliance led to treatment failure	Compliance issues in VA-dual group affecting eradication success
Hu et al., 2022 [50]	Randomized Controlled Trial	110 randomized (154 assessed)	Vonoprazan 20 mg BID + Low-dose (1000 mg BID) or High-dose (1000 mg TID) Amoxicillin for 14 days	H. pylori eradication rate	BID group: 89.1% (ITT), 94.1% (PP); TID group: 87.3% (ITT), 95.9% (PP)	No significant difference between BID and TID amoxicillin groups	No difference in adverse events between groups	Not explicitly mentioned
Qiu et al., 2024 [51]	Randomized Controlled Trial	327 subjects	14-day VA-dual therapy: BM-TID (before meals), AM-TID (after meals), AM-QID (Amoxicillin 750 mg QID after meals)	Eradication rate, adverse events, compliance, antibiotic resistance	BM-TID: 88.1% (ITT), 90.6% (mITT), 90.4% (PP); AM-TID: 89.9% (ITT), 94.2% (mITT), 94.1% (PP); AM-QID: 93.6% (ITT), 99.0% (mITT & PP); AM-QID significantly better than BM-TID	No significant difference between BM-TID and AM-TID; AM-QID significantly better	No significant differences in adverse events among the three groups	Variations in administration timing and compliance could pose challenges
Jiang et al., 2024 [52]	Randomized Controlled Trial	400 patients	Vonoprazan-Amoxicillin dual therapy vs. Bismuth-based quadruple therapy	H. pylori eradication rates and adverse events	Dual therapy: 94.0% (ITT), 97.9% (PP); Quadruple therapy: 87.0% (ITT), 93.0% (PP)	Dual therapy superior in eradication (P = 0.017 ITT; P = 0.022 PP) and fewer adverse events (19% vs. 53%, P < 0.001)	Higher adverse events in quadruple therapy (53%)	Further large-scale validation required before clinical implementation

Vonoprazan triple therapy

VTT, which includes vonoprazan, amoxicillin, and clarithromycin, has emerged as a more effective and intensive treatment for Helicobacter pylori infection compared to traditional therapy using proton pump inhibitors, particularly for clarithromycin-resistant strains. However, its efficacy depends on several factors, such as treatment duration, dosing strategies, and the patient’s resistance pattern. Furthermore, patient compliance and antibiotic resistance must be taken into account when selecting an eradication regimen.

Murakami et al., 2016 [29], showed that vonoprazan-based triple therapy achieved a 92.6% eradication rate, which was superior to lansoprazole-based therapy's 75.9%. Chey et al., 2022 [28], supported these findings by concluding that vonoprazan-based triple therapy achieved an 84.7% eradication rate in non-resistant strains and 65.8%-69.6% in clarithromycin-resistant strains, outperforming lansoprazole-based therapy. In addition, Cheung et al., 2024 [32], concluded the non-inferiority of the vonoprazan-based regimen by reporting an eradication rate of 96.0% with dual therapy and 95.9% with triple therapy, compared to BQT (92.0%), but with fewer side effects.

Despite these promising results, conflicting evidence exists regarding the consistency of vonoprazan-based therapy across different patient populations. Chen et al., 2023 [30], reported no significant difference in eradication rate between vonoprazan-based therapy (77%) and rabeprazole-based therapy (69%) with P > 0.05, suggesting that vonoprazan’s advantage may not be universal. Supporting Chen et al.'s [30] findings, Ratana-Amornpin et al., 2023 [36], found that standard 14-day VTT had a low success rate of 59.3%, necessitating a higher dose or the addition of bismuth to improve efficacy. This suggests that while vonoprazan-based therapy is effective, its performance may be influenced by factors such as antibiotic resistance and regional variation.

Bismuth-Quadruple therapy

BQT, comprising a PPI, bismuth, tetracycline, and metronidazole, is one of the most effective regimens for eradicating *H. pylori*, particularly in regions with high clarithromycin and metronidazole resistance. The bismuth component increases the eradication rate by exerting a bactericidal effect on *H. pylori*.

Wang et al., 2021 [47], reported an eradication rate of 92.7% (per protocol) and 87.1% (intention-to-treat) for tetracycline-based BQT, while furazolidone-based BQT achieved 89.9% (per protocol) and 83.9% (intention-to-treat). Additionally, Hsu et al., 2023 [43], found that BQT (90.2%) was as effective as hybrid therapy (91.5%) and superior to high-dose dual therapy (83.3%), reinforcing its role as a first-line treatment. Yan et al. further validated BQT efficacy, reporting an eradication rate of 89.2% (intention-to-treat) and 91.5% (per protocol), slightly outperforming vonoprazan-amoxicillin dual therapy. However, despite its high efficacy, BQT is often associated with increased adverse effects, which may reduce patient compliance. Liang et al., 2024 [38], found that BQT (89.7%) had a similar eradication rate to vonoprazan-based therapy (90.9%) but was associated with a significantly higher incidence of side effects. Similarly, Zhao et al., 2024 [45], found that BQT reduced gut microbiota diversity and increased pathogenic bacteria, leading to long-term gastrointestinal complications. Furthermore, Macedo et al., 2023 [46], demonstrated that high-dose amoxicillin dual therapy (96.2%) was significantly more effective than BQT (81.4%), particularly in regions with high antibiotic resistance. Its higher side-effect profile necessitates careful consideration of patient tolerance and adherence. Strategies such as shortening treatment duration, optimizing antibiotic selection, and using probiotics may help improve patient compliance while maintaining a high eradication rate.

Amoxicillin therapy

Amoxicillin therapy has emerged as one of the most effective treatment options for *H. pylori* eradication due to its characteristic feature of having fewer side effects, even when administered in high doses and frequency. It is particularly effective in clarithromycin-resistant cases or in patients who cannot tolerate BQT due to its higher incidence of side effects. It also contributes to reducing the pathogenicity of the gut microbiota.

Jiang et al., 2024 [52], found that amoxicillin dual therapy achieved a 97.9% eradication rate (per protocol), outperforming BQT at 93.0% (per protocol), while causing significantly fewer side effects. Qiu et al., 2024 [51], further supported these findings, showing that a 750 mg four-times-daily amoxicillin regimen (99.0% PP) was superior to a three-times-daily regimen (90.6%-94.1% PP). Additionally, Macedo et al., 2023 [46], reported that high-dose amoxicillin dual therapy (96.2%) was significantly more effective than BQT (81.4%), highlighting its potential as a first-line treatment. However, some studies have questioned the consistency of amoxicillin therapy’s efficacy. For example, Hu et al., 2022 [50], found no significant difference between low-dose (89.1%) and high-dose (87.3%) amoxicillin regimens, suggesting that simply increasing the dose may not always enhance efficacy.

Moreover, Hsu et al., 2023 [43], found that high-dose dual therapy (83.3%) was inferior to BQT (90.2%), indicating that while amoxicillin-based regimens are promising, they may not be universally superior to traditional quadruple therapies. Furthermore, Cheung et al., 2024 [32], found that although vonoprazan-amoxicillin dual therapy (96.0%) was comparable to BQT (90.2%), it carried a higher risk of treatment failure due to poor compliance.

Comparative effectiveness of therapies to improve outcomes

The efficacy, safety, and patient compliance associated with VTT, BQT, and Amoxicillin Therapy for Helicobacter pylori infection vary significantly across studies. While each therapy demonstrates promise, regional variations in antibiotic resistance, adverse event profiles, and treatment adherence contribute to inconsistent findings.

Efficacy in eradicating *H. pylori*

Cheung et al., 2024 [32], concluded the non-inferiority of the vonoprazan-based regimen by reporting an eradication rate of 96.0% with dual therapy and 95.9% with triple therapy, compared to BQT at 92.0%, but with fewer side effects.

Yan et al., 2024 [49], found BQT (89.2%) slightly more effective than vonoprazan-amoxicillin dual therapy, though the difference was marginal. Jiang et al., 2024 [52], reported that amoxicillin dual treatment at 97.9% (per protocol) outperformed BQT at 93.0% (per protocol), while causing significantly fewer side effects. Qiu et al., 2024 [51], further supported these findings, showing that a 750 mg four-times-daily amoxicillin regimen (99.0% PP) was superior to a three-times-daily regimen (90.6%-94.1% PP). Macedo et al., 2023 [46], also reported that high-dose amoxicillin dual therapy (96.2%) was significantly more effective than BQT (81.4%). However, Hsu et al., 2023 [43], found that high-dose dual therapy (83.3%) was inferior to BQT (90.2%), indicating that efficacy may be dose-dependent and influenced by regional resistance.

Safety and adverse effects

While BQT is highly effective, it is associated with significantly higher adverse effects compared to VTT and Amoxicillin Therapy.

Liang et al., 2024 [38], found that BQT (89.7%) had a similar eradication rate to vonoprazan-based therapy (90.9%) but was associated with a significantly higher incidence of side effects, including nausea, diarrhoea, and bitter taste.

Similarly, Zhao et al., 2024 [45], found that BQT reduced gut microbiota diversity and increased pathogenic bacteria, leading to long-term gastrointestinal complications. Furthermore, Macedo et al., 2023 [46], demonstrated that BQT was less tolerable than high-dose amoxicillin therapy (96.2% vs 81.4%), raising concerns about adherence.

Conversely, VTT offers a more favourable safety profile than BQT but still causes notable side effects. Cheung et al., 2024 [32], reported that adverse effects were lower in VTT (39%-56%) than in BQT (71%). However, Chey et al., 2022 [28], and Ratana-Amornpin et al., 2023 [36], documented higher rates of nausea, dizziness, and bitter taste with VTT, particularly in patients receiving a clarithromycin-containing regimen.

Amoxicillin-based therapy consistently demonstrates fewer side effects than both BQT and VTT. Jiang et al., 2024 [52], found that adverse effects were significantly lower with amoxicillin dual therapy (19%) compared to BQT (53%). Qiu et al., 2024 [51], confirmed that a higher amoxicillin dose did not increase side effects, making it a tolerable alternative. However, Hu et al., 2022 [50], noted no significant difference in side effects between low-dose and high-dose amoxicillin regimens, suggesting that its safety profile remains stable across doses.

Compliance and clinical applicability

Patient adherence is a key factor in the success of *H. pylori* eradication, and BQT’s high side effect burden often leads to lower compliance. The reporting of dropout rates between therapies shows inconsistent results, although BQT, with its higher side effects and longer treatment duration, often leads to increased patient disenrollment. Amoxicillin-based therapy and VTT demonstrate higher levels of patient adherence because they offer better tolerability along with shorter treatment durations. Research shows that inadequate adherence to specific treatment plans often results in treatment failure, despite being documented in published studies.

Cheung et al., 2024 [32], and Yan et al., 2024 [49], noted that poor adherence to BQT contributed to treatment failure, emphasising the need for strategies to improve tolerability. VTT has better compliance due to its shorter treatment duration and improved tolerability. Waqar et al., 2024 [35], found that 7-day vonoprazan therapy (95.1% eradication) was not inferior to 14-day esomeprazole therapy (93.1%), making it a cost-effective and convenient option. However, Ratana-Amornpin et al., 2023 [36], observed low compliance in standard-dose vonoprazan therapy, suggesting that a higher dose or bismuth supplementation may be necessary in some cases.

Amoxicillin therapy offers the highest compliance due to its minimal side effects but requires strict adherence to a frequent dosing schedule. Jiang et al., 2024 [52], and Qiu et al., 2024 [51], reported high adherence (94%-99%) in amoxicillin-based therapy. Still, Cheung et al., 2024 [32], highlighted that poor compliance contributed to treatment failure in some vonoprazan-amoxicillin regimens, especially when patients deviated from the prescribed dosing schedule.

Discussion

The current study evaluated the efficacy of VTT, BQT, and Amoxicillin Therapy for Helicobacter pylori infection. A study conducted by Jung et al., 2017 [53], assessed the efficacy of VTT compared to proton pump inhibitors. The analysis included 10 studies with 10,644 patients. The eradication rate for VTT was 87.9%, significantly higher than that of PPI-based therapy (pooled risk ratio = 1.19, 95% CI: 1.15-1.24), supporting the current study’s findings, which indicate that VTT achieved an eradication rate of 88.2% (intention-to-treat analysis).

Similarly, Lyu et al., 2019 [54], conducted a meta-analysis comparing VTT to proton pump inhibitors. The analysis included three studies with 897 patients. The eradication rate for VTT was 91.4%, significantly higher than that of PPI-based therapy (odds ratio = 3.68, 95% CI: 1.87-7.26), supporting the current study’s findings. While the eradication rate in Lyu et al.’s study was higher than the current study's rate of 88.2% (intention-to-treat analysis), this may be attributed to differences in sample population and resistance patterns across regions.

Lyu et al. (2019) also concluded that VTT had lower adverse effects (32.7% vs. 40.5%; OR = 0.71, 95% CI: 0.53-0.95). This finding aligns with the current study, which found that 30.8% of patients experienced adverse effects, with mild gastrointestinal symptoms being the most common.

Guo et al., 2021 [55], conducted a meta-analysis comparing the efficacy and safety of BQT and concomitant therapy (CT) for treating *H. pylori* infection. The analysis included ten studies. The eradication rate was 84.6% for BQT and 82.95% for CT (OR = 1.14, 95% CI: 0.94-1.38; P = 0.19). In the per-protocol analysis, BQT had a higher eradication rate (92.4% vs. 90.1%; OR = 1.32, 95% CI: 1.00-1.73; P = 0.05). These eradication rates support the current study’s findings, which reported an eradication rate of 85.5%.

Another meta-analysis conducted by Ouyang et al., 2022 [56], compared susceptibility-guided therapy (SGT) with BQT. Ouyang et al. found slightly better outcomes with susceptibility-guided treatment (92.4% eradication rate) (OR = 1.35, 95% CI: 1.13-1.61; P < 0.001), suggesting that individual antibiotic resistance testing may improve BQT efficacy. The findings imply that tailored therapy based on antibiotic susceptibility may enhance treatment outcomes.

The eradication rate of 92.4% for SGT supports the current study's findings, which reported eradication rates ranging from 87.1% to 92.7%. Although the current study did not specifically address SGT, the findings support the broader implication that antibiotic resistance significantly affects BQT efficacy.

The current study also found that 38.2% of BQT-treated patients experienced adverse events, with nausea and diarrhoea being the most frequent side effects, potentially affecting patient compliance. Guo et al., 2021, supported these findings by reporting that the overall incidence of adverse events was similar between the BQT and CT groups.

The current study finds that Amoxicillin Therapy has an eradication rate of 86.1%. Yang et al., 2019 [57], supported this finding by conducting a systematic review focusing solely on amoxicillin-based therapy. They reported that amoxicillin dual therapy achieved a 97.9% eradication rate, outperforming BQT, which had a 93.0% eradication rate, with significantly fewer adverse effects (19% vs. 53%). These findings suggest that high-dose amoxicillin-based therapy may be a promising alternative to traditional regimens, particularly in regions with high antibiotic resistance or among patients who experience adverse effects with other therapies. The current study also finds that the incidence of adverse events was 28.5% in amoxicillin-based therapy, the lowest among the three therapies studied. Yang et al., 2019, noted that amoxicillin-based therapy had fewer side effects than BQT. These findings align with previous systematic reviews, reinforcing that amoxicillin-based treatment has the best safety profile while maintaining efficacy similar to BQT.

Despite its several advantages, the study has some limitations. Different antibiotic resistance patterns may obscure the study findings, and there are limitations regarding the effect of drugs on different population groups. Future studies should be conducted on a larger scale, comprising diverse populations, to increase the applicability and generalizability of findings. The most effective treatment regimen should be tailored to individuals to achieve optimal outcomes and reduce the development of antibiotic resistance.

## Conclusions

The management of *H. pylori* infection is a dynamic process. As resistance patterns evolve and new treatments are developed, it is important for healthcare providers to stay informed about emerging options. Personalized treatment, taking into account patient preferences, tolerability, and concerns about antibiotic resistance, will likely continue to offer the best chance for successful eradication. Medical practitioners should utilize BQT when dual resistance rates against clarithromycin and metronidazole are high. VTT proves best in regions with low or moderate clarithromycin resistance. High-dose Amoxicillin therapy works best when clarithromycin resistance dominates in these areas. The additional definition makes our conclusion better align with treatment approaches that consider resistance patterns. Ultimately, the goal is to select the most suitable therapy for each patient, ensuring both the effectiveness and comfort of the treatment. The review concluded that BQT remains a highly effective first-line therapy for *H. pylori* infection, but the side effect burden limits its applicability. VTT offers a superior eradication rate with improved tolerability, making it an effective treatment option. However, high doses of amoxicillin emerge as the safest and most effective treatment option, especially where clarithromycin resistance is prevalent. Tailoring therapy to specific patient needs can improve eradication success, reduce adverse effects, and ultimately enhance patient satisfaction in the management of *H. pylori* infection.
